# IL-6/IL-12 superfamily of cytokines and regulatory lymphocytes play critical roles in the etiology and suppression of CNS autoimmune diseases

**DOI:** 10.3389/fimmu.2025.1514080

**Published:** 2025-03-06

**Authors:** Manoj Kumar Yadav, Samara P. Singh, Charles E. Egwuagu

**Affiliations:** Molecular Immunology Section, Laboratory of Immunology, National Eye Institute (NEI), National Institutes of Health (NIH), Bethesda, MD, United States

**Keywords:** cytokines, uveitis, multiple-sclerosis, EAU, EAE, JAK/STAT, exosomes, nanobody

## Abstract

Cytokines influence cell-fate decisions of naïve lymphocytes and determine outcome of immune responses by transducing signals that regulate the initiation, intensity and duration of immune responses. However, aberrant regulation of physiological levels of cytokines contribute to the development of autoimmune and other inflammatory diseases. The Interleukin 6 (IL-6)/IL-12 superfamily of cytokines have a profound influence on all aspects of host immunity and our focus in this review is on the signaling pathways that mediate their functions, with emphasis on how this enigmatic family of cytokines promote or suppress inflammation depending on the physiological context. We also describe regulatory lymphocyte populations that suppress neuroinflammatory diseases by producing cytokines, such as IL-27 (i27-Breg) or IL-35 (i35-Breg and iT_R_35). We conclude with emerging immunotherapies like STAT-specific Nanobodies, Exosomes and Breg therapy that ameliorate CNS autoimmune diseases in preclinical studies.

## 1 Introduction

Cytokines are categorized into distinct families based on structural homology of their proteins or receptors as well as their functional characteristics. They include interleukins, chemokines, interferons, and tumor necrosis factor (1–3). In the internal milieu of an organism, a lymphocyte or myeloid cell type can secrete cytokines that influence its function or the behavior of surrounding cells in an autocrine or paracrine manner. Upon binding to cognate receptors on target cells, the cytokine signal is transmitted to the nucleus through a variety of intracellular proteins including, the 7-member signal transducer and activator of transcription (STAT) family of transcription factors that activate or repress gene transcription in the target cell (4, 5). In the immune system, cytokines of the IL-6 and IL-12 (IL-6/IL-12) superfamily play critical roles in instructing the naïve lymphocyte to differentiate into T-helper 1 (Th1), Th2 or Th17 subset, regulatory T cell (Treg) subsets (i10-Treg, iT_R_35, Tr1) or regulatory B cell (Breg) subsets (i10-Breg, i27-Breg, i35-Breg) (6–9). On the other hand, TNF (tumor necrosis factor), IFN-γ (interferon gamma) or TGF (transforming growth factor) family of cytokines mediate effector functions that influence the behavior or activities of target cells, while chemokines guide the trafficking of lymphocytes to sites where they mediate their effector functions. Importantly, physiological levels of cytokines are, in turn, under stringent control by intracellular negative-feedback regulators. Among these, suppressors of cytokine signaling (SOCS) proteins play the predominant role by inhibiting JAK(Janus Kinase)/STAT pathway, thereby preventing unbridled lymphocyte activation that can cause autoimmune diseases (10, 11). Cytokines that activate JAK/STAT pathway have profound influence on naïve T cell developmental decisions and regulate critical lymphocyte effector functions, that determine whether the immune response would be protective or induce autoimmunity. In this review, we have summarized the roles IL-6/IL-12 superfamily cytokines play in activating protective immunity or mediating the vicious cycles of relapsing-remitting inflammation, characteristic of neuroinflammatory diseases like multiple sclerosis or the sight threatening intraocular inflammatory disease (uveitis), which are important public health diseases.

## 2 The IL-6 family of cytokines

### 2.1 Immunobiology of IL-6 family cytokines

**Figure 1:**
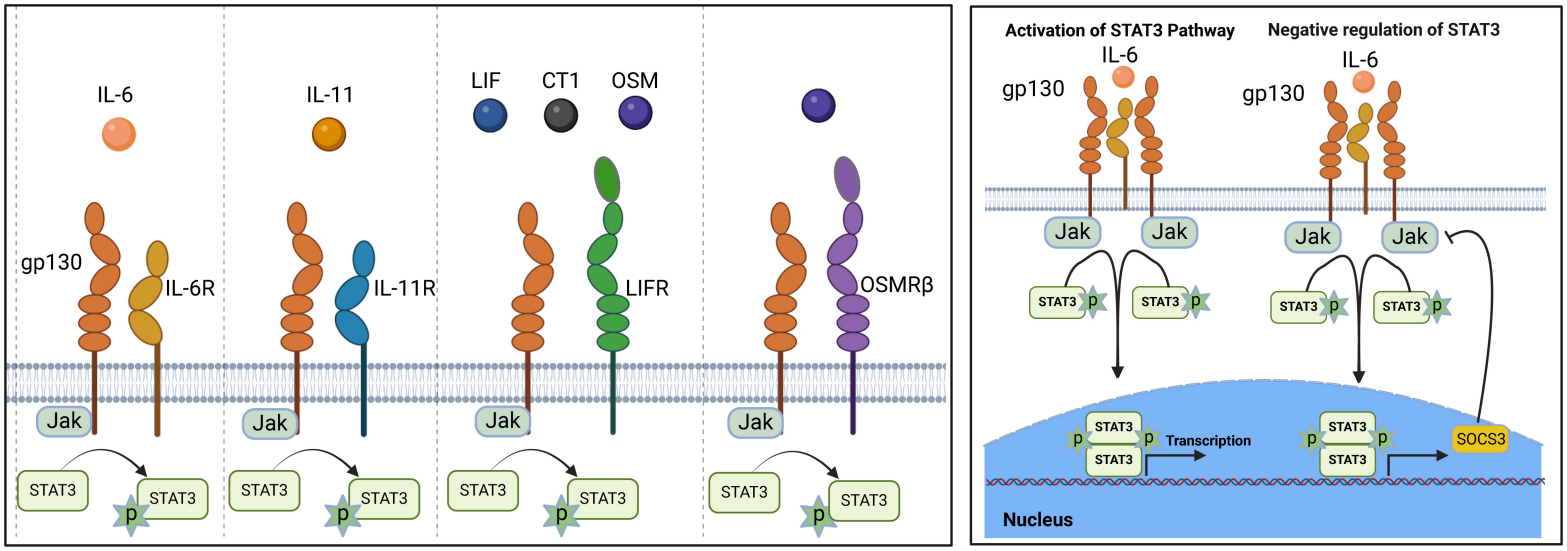
IL-6 signaling and regulation by suppressor of cytokine signaling (SOCS): Members of the IL-6 family cytokine share signal-transducing gp130 (IL-6Rβ) and are under negative feedback regulation by SOCS1 and SOCS3.

**Figure 2:**
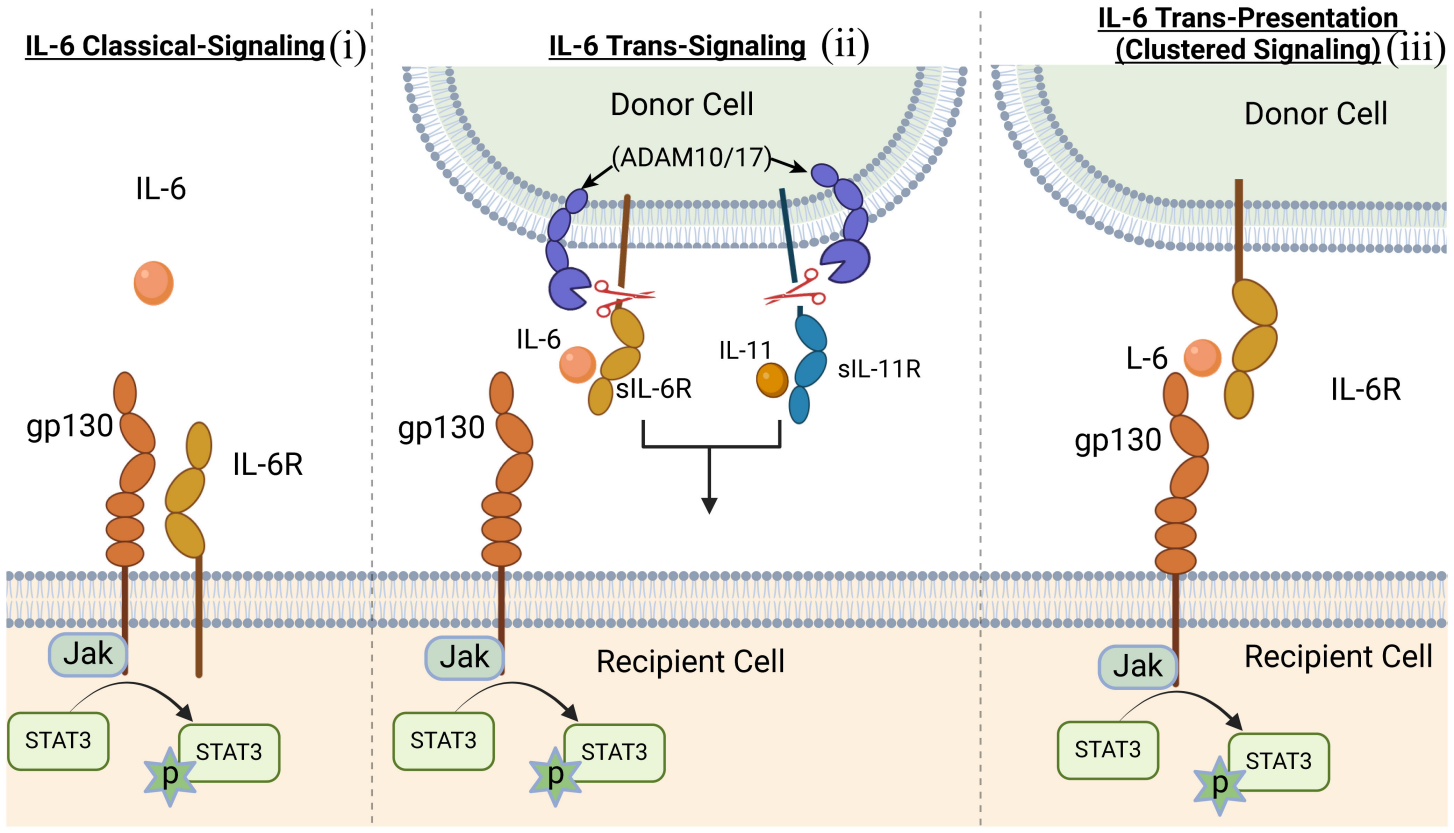
Scheme of the three distinct forms of IL-6-mediated signaling mechanisms. (i) classic IL-6 signaling; (ii) IL-6 trans-signaling; (iii) IL-6 trans-presentation or cluster signaling.

IL-6 is the prototypical member of the IL-6 family, characterized by common usage of the signal transducing transmembrane glycoprotein 130 (gp130 or IL-6Rβ) (12, 13). The family also includes IL-11, ciliary neurotrophic factor (CNTF), oncostatin M (OSM), leukemia inhibitory factor (LIF), cardiotrophin 1 (CT-1) and cardiotrophin-like cytokine factor 1 (CLCF1) (14). Members of the IL-6 family are pleiotropic cytokines produced by a wide variety of immune as well as non-immune cells and they play crucial roles in a variety of autoimmune diseases. In addition to their roles in hematopoiesis and maintenance of immune homeostasis, they regulate diverse cellular process that affect embryonic development, cognition, intermediary metabolism and ageing. They signal through a non-signaling alpha (α) receptor subunit (IL-6R, CNTFR, LIFR, OSMR or IL-11R) which binds the cognate cytokine and the cytokine:α-receptor complex then interacts with gp130 (IL-6Rβ) to form the high-affinity homodimer receptor complex (15). Signaling through gp130 homodimer results in the phosphorylation of receptor-associated Janus kinases (JAK1, JAK2) that preferentially recruit STAT3 to the cytokine receptor complex for activation via tyrosine phosphorylation (16) (**Figure 1**). Another important feature of IL-6 family cytokines is their propensity to generate soluble receptors that derive from shedding extracellular portion of their α or β receptors through limited proteolysis by metalloproteinases such as ADAM17 (a disintegrin and metalloproteinase domain-containing protein 17) and ADAM10 (17, 18). The soluble α receptor (e.g. sIL6R) retains capacity to bind cognate cytokine (e.g. IL-6) *in vivo* to form receptor-ligand complexes that interact with membrane-bound gp130 to activate cells. Thus far three distinct forms of IL-6-mediated signaling have been described: (i) The classic IL-6 signaling where IL-6 binds membrane-bound IL-6R (mIL-6R), initiates signaling by interacting with gp130 homodimers. This results in activation of JAK1/JAK2, followed by tyrosine-phosphorylation of STAT3 and subsequent translocation of pSTAT3 homodimers into the nucleus; (ii) IL-6 trans-signaling is suggested to broaden the variety of cells responsive to IL-6, IL-11 or possibly CNTF. In IL-6 trans-signaling, IL-6 and sIL-6R complexes initiate signal transduction by recruiting and binding membrane-bound gp130 (mgp130); (iii) IL-6 trans-presentation or cluster signaling is another receptor engagement mechanism where IL-6-mIL6R complexes on a cell activate gp130-gp130 homodimers on a neighboring cell, resulting in enhanced lymphocyte activation or activation of target cells that lack specific receptors for IL-6 cytokines. Suppressor of cytokine signaling-1 (SOCS1) and SOCS3 are important negative-feedback regulators of IL-6 family cytokines that prevent excessive cytokine activation that can lead to tissue damage and disease. SOCS3 functions by targeting gp130 receptor, JAK homology domains and STATs for ubiquitin-mediated degradation in the proteasome (19, 20) (**Figures 1**, **2**).

### 2.2 Role of IL-6 in inflammatory and autoimmune diseases

With regards to the role of IL-6 in human diseases, it is notable that blood levels of IL-6 in a healthy human is 1-5 pg/ml and can increase to 1000 folds in some inflammatory conditions and up to several µg/ml during sepsis (21). Pre-clinical studies have also implicated IL-6 in impaired immune responses, arthritis, colitis, and multiple sclerosis and activation of mucosal T cells induced by IL-6-sIL-6R exacerbates Crohn disease, a chronic inflammatory disease of the gastrointestinal tract chronic intestinal inflammation (22–26). Thus, elevated level of IL-6 is pathognomonic sign of inflammation and drugs like Tocilizumab (Actemra) that target IL-6 and/or IL-6R are effective and approved for the treatment of autoimmune diseases including rheumatoid arthritis (27). Therapeutic targeting of components of the cytokine receptor signaling complex utilized by IL-6-like cytokines including gp130 or the proinflammatory IL-6 trans-signaling pathway are also effective and represent new avenues for drug discovery. Thus, treatments approved or undergoing clinical trials in the USA include cytokine-blocking antibodies (Abs), receptor-blocking antibodies, agonist cytokine-antibody complexes, small-molecule Jak inhibitors (Jakinibs), STAT-binding inhibitory peptides (8), small-molecule STAT inhibitors (28). Although JAK inhibitors are in use for the treatment of rheumatoid arthritis and other inflammatory diseases (29, 30), it must be emphasized that JAKs do not selectively inhibit IL-6 signaling pathway and off-target effects may interfere with other cytokines or growth factors that utilize JAK/STAT pathway (28). Nonetheless, because IL-6 cytokines predominantly utilize STAT3 pathway and the pathogenic Th17 cells that mediate CNS autoimmune diseases require STAT3 (31, 32), there is significant interest in developing therapies that target STAT3 pathway. These emerging therapies include SOCS mimetics and nanobodies that suppress disease in mouse models of uveitis and multiple sclerosis (33–35).

Similar to the relatively low amount of IL-6 in human blood that increases in disease state (1-5 pg/ml), the level of soluble IL-6 receptor (sIL-6R) is around 40-75 ng/ml and can increase by 2-10 folds during inflammatory diseases. In contrast, sgp130 levels in healthy individuals is high (~250-400 ng/ml), remains unaltered during inflammation and this has led to the suggestion that endogenous sgp130 serves to restrain or buffer systemic IL-6 trans-signaling activities (21, 36). These and other experimental findings indicate that IL-6 trans-signaling is responsible for the proinflammatory activities of IL-6 while the classic signaling mechanism mediated by direct interaction of IL-6 and mIL6R promotes anti-inflammatory activities of IL-6. In this context, the monoclonal antibody, Olamkicept (soluble gp130Fc or sgp130Fc), that specifically inhibits IL-6 trans-signaling without interfering with classic IL-6 signaling, is effective treatment for inflammatory bowel disease (IBD). Its clinical effectiveness correlates with suppression of STAT3 phosphorylation and transcriptional changes in the inflamed mucosa (37, 38). Compared to other antibody treatments, very low amount of sgp130 is required to prevent fatal sepsis in mice (39), underscoring the merit of blocking of IL-6 trans-signaling. Olamkicept is well tolerated and clinically desirable because of practicality of limiting the pharmacological dosing regimen.

## 3 IL-12 family cytokines

**Figure 3:**
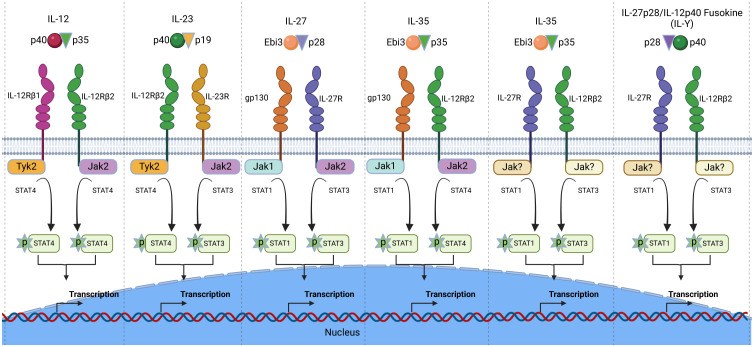
The IL-12 cytokine family and its heterodimeric receptors. The family is comprised of heterodimeric cytokines that share receptor components and mediate biological functions through activation of the JAK/STAT signal transduction pathway. The Janus kinases (Jaks) and STATs utilized by each IL-12 cytokine is depicted.

**Figure 4:**
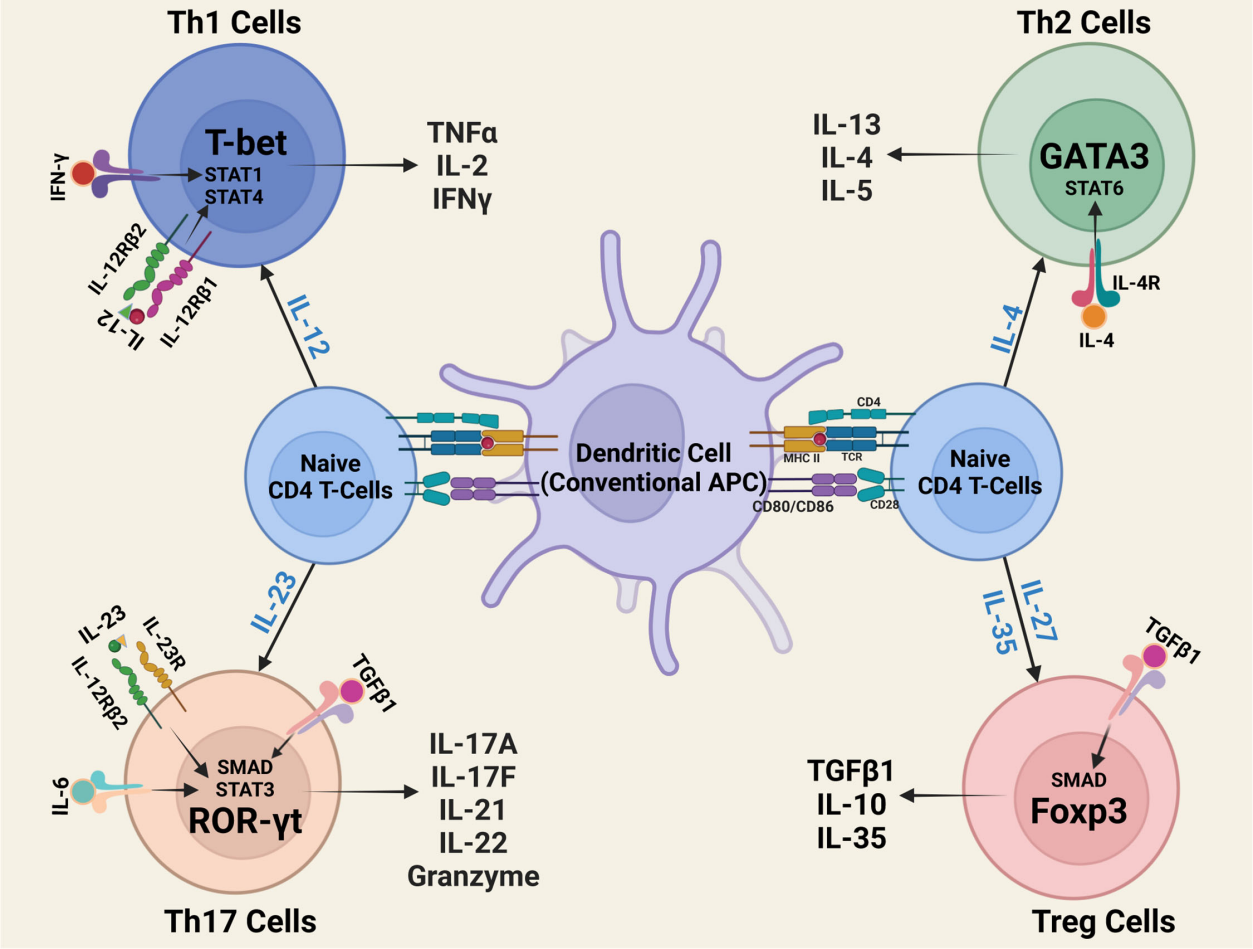
Outline of CD4^+^ T-helper-cell differentiation. During antigen presentation in the lymph nodes, dendritic cells (DCs) and other antigen-presenting cells (APCs) secrete IL-12 family cytokines that instruct naïve T cells to differentiate to Th1, Th2, Th17 or Treg subset.

Interleukin-12 type cytokines (IL-12 family) are comprised of two subunits, an α-subunit protein with a helical structure similar to type 1 cytokines (e.g. IL-6), and a signal-transducing β-subunit structurally related to the soluble IL-6 receptor (IL-6Rα). In essence, they are heterodimers consisting of a cytokine and a soluble cytokine receptor. The family includes IL-12 (IL-12p35/IL-12p40), IL-23 (IL-23p19/IL-12p40), IL-27 (IL-27p28/Ebi3), IL-35 (IL-12p35/Ebi3), IL-39 (IL-23p19/Ebi3) and the novel fusokine, provisionally denoted IL-Y (IL-27p28/IL-12p40) (40–43). IL-12 cytokines have emerged as important regulators of host immunity (**Figure 3**). They play critical roles in the developmental decisions that determine the repertoire of lymphocyte subsets participating during an ensuing immune response. Their effects on host immunity derives from the fact that transcription of genes that encode the α and β subunits are the immediate targets of IL-12 cytokines secreted by activated Ag-presenting dendritic cells (APC) during Ag-priming. Thus, the naïve lymphocytes can differentiate into pro-inflammatory or immune-suppressive subsets depending on the TLRs that engage the APCs and the predominant IL-12 cytokine type in the lymph nodes required for transcriptional activation of specific α or β gene. For example, Gram-negative bacteria engage TLR4 on APCs, induce secretion of IL-12 and skews the immune response towards the Th1 developmental pathway. On the other hand, activation of TLR2 by Gram positive bacteria or fungi increases the secretion of IL-23, skewing the response towards Th17. In contrast to the proinflammatory activities elicited when IL-12 or IL-23 is the predominant cytokine produced during Ag-priming, sustained TLR signaling downstream of MyD88 and TRIF adaptor molecules increase transcription of IL-27p28 and IL-12p35 subunits required for secretion of IL-27 or IL-35 and expansion of regulatory lymphocytes (44). However, effects of IL-27 are complex as IL-27 can induce expansion of proinflammatory or immunosuppressive subsets depending on the physiological context. On the other hand, intense inflammation induces production of IL-35, which promotes differentiation of regulatory lymphocytes that suppress inflammation and autoimmune diseases (45–47) (**Figure 4**). Thus, vaccines containing adjuvants that induce IL-12 and IL-23 would be more effective in preventing infectious diseases while IL-27 and IL-35 suppress autoimmune diseases. It is notable that the two subunits of IL-12 or IL-23 form covalently linked heterodimers while the α and β chains of IL-27 or IL-35 are not covalently Therefore it has been technically difficult to isolate or produce biologically active heterodimeric IL-27 or IL-35 and this has hampered therapeutic use of these immune-suppressive cytokines. In fact, biologically active heterodimeric IL-35 or IL-27 is not commercially available, although both cytokines are now available as recombinant fusion proteins.

### 3.1 IL-12 and IL-23: roles in immunity and pathology

IL-12 was identified and purified from supernatant fluid of EBV transformed B lymphoblastoid cell line in 1989 (40, 41). The 70kDa heterodimeric glycoprotein, comprised of IL-12p40 and IL-12p35, was initially designated as NK cell stimulatory factor (NKDSF). In 2000, the IL-12p19 protein was identified on the basis of its homology with IL−6 and was demonstrated to dimerized with the IL-12p40 subunit to form IL-23 (48). Both cytokines were subsequently found to exhibit similar as well as distinct biological activities (49). However, mice deficient in IL-12p40 deficient mice were subsequently shown to be resistant to several mouse autoimmune diseases including experimental autoimmune encephalomyelitis (EAE), experimental autoimmune uveitis (EAU) and T cell mediated colitis (48). These findings prompted re-evaluation of functions previously attributed to IL−12 based on IL-12 neutralization experiments, deletion of IL-12p40 or its receptor (IL−12Rβ1). These investigations revealed that IL−23-driven Th17 cells, rather than the Th1 subset, mediates autoimmune or infectious diseases previously attributed to IL-12-induced Th1 subset (48). Although EAU was also thought to be a Th1-mediated disease (50), mice with targeted deletion of STAT3 were found to be resistant to this CNS autoimmune disease of the retina. Although these mice with loss of STAT3 in CD4^+^ T cells also lack Th17 cells they exhibit exaggerate expansion of Th1 cells, providing direct evidence that IFN-γ produced by Th1 cells are protective while the IL-17 producing Th17 cells are responsible for inducing ocular pathology in the EAU model (31, 51).

### 3.2 Immunobiology of IL-27 and IL-35

Ebi3 was identified in 1996 from a subtractive hybridization screen of genes expressed in Epstein-Barr virus (EBV) transformed B cell lines (52). It is structurally homologous to IL-12p40 and contains no membrane anchoring motifs, suggesting that it is a secreted protein (52). Given the homology of Ebi3 to IL-12p40, IL-12p35 was posited as a potential pairing partner for Ebi3 and this was confirmed by co-expression studies identifying the IL-12p35:Ebi3 heterodimer as IL-35 (53, 54). It is also of note that the Ebi3 protein has 3 methionine and 4 cysteine residues while IL-12p35 has 10 methionine and 7 cysteine residues and this may account for the interaction between the two subunits. Similar to IL-35, discovery of IL-27 derived from *in silico* and biochemical approaches using genomic databases of α-helical cytokines of the IL-6 family. These studies led to the identification of IL-27p28 (also called IL-30) and its pairing with EbI3 to form the heterodimeric IL-27 cytokine (55). The preferential expression of IL-27 and IL-35 by activated lymphocytes and myeloid cells (DC, monocytes and macrophages), underscore the broad roles these cytokines play in regulating immunity. Despite the significant therapeutic interest in IL-27 and IL-35, mechanisms that regulate production of IL-27 or IL-35 cytokine is not well understood. Unlike proinflammatory members of the IL-12 family cytokines (IL-12 and IL-23) that are secreted and function as disulfide-linked heterodimers, IL-27 and IL-35 are non-covalently linked IL-27p28:Ebi3:IL-12p35:Ebi3 heterodimers, respectively. Important unresolved issues relating to the immunobiology of IL-27 and IL-35 include physiological cues that initiate the association of their two subunit proteins, how stability of the heterodimeric cytokine is maintained and mechanism that regulate bioavailability of the native IL-27 or IL-35 under physiological conditions. Both cytokines share the common Ebi3 subunit which encodes a single signal-peptide that is cleaved concomitant with translocation into the lumen of the endoplasmic reticulum, suggesting that it is a secreted protein. In contrast, IL-12p35, in the mouse species, contains two tandem signal-peptides and do not conform to the classic co-translational model of signal-peptide removal. Thus, it has been predicted that IL-12p35 is retained in the endoplasmic reticulum or distal Golgi as a transmembrane protein (56, 57).

### 3.3 IL-35 and IL-27: regulators of immunity during CNS autoimmune diseases

The central nervous system (CNS) comprises of immune privilege tissues that include the brain, spinal cord and retina and these CNS tissues are sequestrated from the peripheral immune system by the blood-brain-barrier (BBB), blood-retina barrier (BRB) and the neurovascular unit (NVU) (10, 11). The immune system of the CNS is unique and exquisitely regulated to confer adequate protection from pathogens while calibrating intensity of the response to avoid collateral damage of the terminally differentiated neurons required for cognition and vision (58). While much is known about the roles played by Th1 and Th17 cells in fueling the vicious cycles of relapsing-remitting inflammation that characterizes multiple sclerosis (59) and the sight-threatening intraocular inflammatory diseases or uveitis (51, 60), less is known about the protective roles B cells play. Here we discuss the roles B cells play in suppressing CNS autoimmune diseases with a particular focus on IL-27-producing and IL-35-producing regulatory B cells (Bregs) and IL-35 containing exosomes (i35-exosomes) and IL-27-exosomes (i27-exosomes) that they secrete.

### 3.4 IL-35-producing Bregs

Studying the role lymphocytes play in autoimmune diseases led to discovery of IL-35-producing Breg cells (i35-Bregs) that suppress neuroinflammatory diseases and also IL-35-producing Treg cells that maintain immune tolerance and promote tumor T cell exhaustion (47, 61–64). These observations spurred interest in developing i35-Breg immunotherapy for CNS autoimmune diseases (47). Despite the role of IL-35 in suppressing inflammatory diseases and inhibiting cytotoxic CD8^+^ anti-tumor T cells that prevent tumor metastasis (47, 61, 64), gaps in mechanistic understanding of how IL-35 mediates immunosuppression have prevented its therapeutic use. On the other hand, recent reports have suggested that IL-12p35 and Ebi3 are not necessarily secreted as heterodimeric IL-35 cytokine, but that these IL-12p35 and Ebi3 subunits are independent anti-inflammatory cytokines (45, 46, 65). This has raised interest in understanding the immunobiology of the i35-Breg cell and the mechanisms that underlie its immunosuppressive activities.

Thus far, there is no unique marker or set of markers that exclusively identify the i35-Breg cell and designation of a i35-Breg cell is mainly based on production of IL-35 as well as the capacity to suppress inflammation either *in vivo* or *in vitro*. Although the capacity to produce immune-suppressive cytokines by i35-Bregs is firmly established, mechanisms that mediate IL-35 production by B cells is not well understood. Interferon regulatory factor-4 (IRF-4) and IRF-8 are expressed in developing B cells and play critical roles in B cell development and effector functions (66, 67). Neither IRF-4 nor IRF-8 binds DNA strongly, and they depend on the basic leucine zipper transcription factor ATF-like (BATF) to recruit them to promoter sites of genes they regulate (66). These crippled transcription factors bind competitively to BATF and concentration-dependent competition between them control cell-fate decisions of the differentiating B cell (67, 68). In activated B cells, BATF dimerizes with IRF-4, resulting in the recruitment of BATF-IRF-4 complex to AP1-IRF-composite elements (AICEs) of the *Il12a* locus, suggesting the involvement of BATF-IRF-4-AICE complex in regulating the IL-35 transcriptional programs of Breg cells (69). In fact, *Irf4* ^fl/fl^Cd19^+/Cre^ B cells with loss of IRF-4 in B cells are defective in generating i35-Bregs, underscoring the role of IRF-4 in skewing of B cells toward the i35-Breg phenotype. Therapeutic importance of i35-Bregs is suggested by pre-clinical studies showing that 500,000 *ex-vivo* generated i35-Bregs is sufficient to suppress uveitis or encephalomyelitis in the EAU or EAE mouse model, respectively (47, 70). Most importantly, the i35-Breg cells were administered 4 days after EAU or EAE induction. Mitigation of disease in mice with ongoing disease indicates that i35-Breg immunotherapy can be used to treat individuals with established autoimmune disease.

### 3.5 IL-27-producing Bregs

An innate-like IL-27 producing population of natural regulatory B-1a cell (i27-Breg) was recently identified in the peritoneal cavity and human umbilical cord blood (8). Most Breg cells, including the i35-Breg cells, derive from the B-2 lymphocyte lineage which are the most abundant B cells in mammals that develop in the bone marrow and reside mainly in the spleen. In contrast, B-1 lymphocytes are the earliest B cells to arise during development from the fetal yoke sac and they reside mainly in the peritoneal cavity and cord blood (71). Similar to the role played by BATF and IRF transcription factors in the transcriptional program of i35-Breg cells, BATF dimerizes with IRF-8, resulting in the recruitment of BATF-IRF-8 complex to AICEs of *il27p28* locus, underscoring involvement of BATF-IRF-8-AICE complex in regulating i27-Breg transcriptional program (8). *Irf8* ^fl/fl^Cd19^+/Cre^ mice that do not express IRF-8 are defective in generating i27-Bregs, indicating that IRF-8 may skew B cells toward the i27-Breg phenotype. Similar to i35-Breg cells, adoptive transfer of 500,000 i27-Breg cells to mice with EAU or EAE suppressed and ameliorated uveitis and encephalomyelitis respectively, in pre-clinical studies (8).

## 4 Emerging immunotherapy for CNS autoimmune diseases

### 4.1 Exosome immunotherapy

The i27-Bregs and i35-Bregs secrete exosomes that contain IL-27 (i27-Exosomes) or IL-35 (i35-Exosomes), respectively (70, 72). These nanosized extracellular vesicles range from 50 nm to 150 nm as determined by electron microscopy. Importantly, their small sizes allow them to cross the blood-brain-barrier or blood-retina-barrier, making them ideal vehicles for therapeutic delivery of immunosuppressive cytokines like IL-27 or IL-35 into the CNS. Besides B cell-derived exosomes, Treg cells that produce IL-35 (iT_R_35) also secrete IL-35-coated extracellular vesicles and they promote infectious tolerance (73). In preclinical studies, *ex-vivo* generated i27-Exosomes or i35 Exosomes were evaluated as potential stand-alone therapy for uveitis in the EAU model. The i27-Exosomes or i35-Exosomes ameliorated uveitis by suppressing expansion of pathogenic Th17 cells while inducing expansion regulatory lymphocytes that produce immunosuppressive cytokines including IL-10, IL-27 and IL-35 (70). Importantly, these exosomes also propagate infectious-tolerance mechanisms that suppressed uveitis by upregulating the expression of checkpoint proteins on the plasma membrane of autoreactive T-cells. These proteins that are associated with T-cell exhaustion or anergy include PD-1 (programmed cell death protein 1), LAG-3 (lymphocyte-activation gene 3), CTLA-4 (cytotoxic T-lymphocyte associated protein).

### 4.2 Camelid-derived STAT-specific nanobody immunotherapy

CNS diseases result from dysregulation of intricate biochemical pathways activated and utilized by the myriads of neurons, microglia and other myeloid cell types that mediate critical functions of the brain, spinal cord and neuroretina (58). Cytokines, growth factors, hormones and neurotransmitters that regulate and coordinate the activities of these diverse cell types in the CNS are in turn under stringent regulation by transcription factors that control the activation or repression of genes involved physiological processes of CNS tissues. Thus, a major challenge in the treatment of CNS diseases derives from the fact that transcription factors are undruggable because they are intracellular proteins, and they are not amenable to antibody therapy. This is particularly relevant in context of CNS autoimmune diseases such as uveitis and multiple sclerosis. The transcription factor STAT3 is implicated in uveitis because mice with loss of STAT3 in CD4^+^ T cells do not develop EAU (31). In multiple sclerosis, IFN-γ-producing Th1 cells induce ascending paralysis and promote recruitment of inflammatory cells into the thoracolumbar spinal cord. On the other hand, IL-17-producing Th17 cells mediate atypical MS, characterized by cerebellar ataxia (74–76). Although Jak inhibitors are effective and approved by the FDA, toxicity considerations limit their use (28–30).

Camelids (alpaca, llama, camel, vicuna and guanaco) produce non-canonical immunoglobulins comprised of a single variable heavy chain (VHH) and no light chain (77). In contrast to conventional antibodies which are 150kDa (~15nm), VHH miniature antibodies (also called Nanobodies) are only 15kDa (~2.5nm). Therapeutic interest in Nanobodies in CNS autoimmune diseases derives from the fact that they readily enter cells by transcytosis and into the CNS after inflammation-induced break-down of blood-brain-barrier or blood-retinal-barrier (BRB) (77). A novel Nanobody (SBT-100) was recently produced by immunization of Dromedary camels (Arabian camel) with human STAT3. Extensive characterization of SBT-100 revealed that it is specific to the highly conserved SH2 domain shared by all STAT proteins. In the EAU model SBT-100 inhibited the expansion of pathogenic Th17 cells and suppresses uveitis in mice (35). Because STAT1 and STAT3 are required for the development of naïve T cells into Th1 and Th17 lymphocyte, pre-clinical studies examined whether simultaneous inhibition of STAT1 and STAT3 with SBT-100 would be effective therapy for multiple sclerosis. The SBT-100 Nanobody prevented development of encephalomyelitis by inhibiting the recruitment of CD4^+^ T cells into the brain and spinal cord and diminished the capacity of encephalitogenic T cells to transfer EAE to naïve syngeneic mice (78). The success of SBT-100 in suppressing uveitis or encephalomyelitis in mice provides a template for use of SBT-100 Nanobody in modulating undruggable transcription factors such as STAT1 and STAT3 (35, 78). Camelid-derived Nanobodies are not immunogenic in humans because of their high sequence homology with human antibodies and the small size facilitates transport across the BBB and BRB during inflammation providing important advantages for therapeutic use in CNS diseases.

## 5 Conclusion

Significant progress has been made in cytokine research, leading to identification of novel cytokines and establishment of a cytokine classification system based their structure, receptor usage and function. Progress in this field has also led to a better understanding of cytokine signaling pathways that regulate diverse biological processes including hematopoiesis, embryonic development, cognition, intermediary metabolism and ageing. In this review we have highlighted cytokines of the IL-6/IL-12 superfamily with focus on IL-6, IL-12 and IL-23 that are implicated in etiology of rheumatoid arthritis, multiple sclerosis, uveitis and Crohn’s disease. Mice deficient in various aspects of the JAK/STAT signal transduction pathway utilized by IL-6/IL-12 cytokines have also provided mechanistic insights on how proinflammatory T cell subsets contribute to the development of autoimmune diseases. However, the role of B lymphocytes is still under-studied.

Here, we have highlighted two newly identified regulatory B cell populations that suppress autoimmune diseases. The innate-like i27-Bregs derive from the B-1 lymphocyte lineage while i35-Bregs derive from the B-2 lineage and both Breg types secrete immunosuppressive exosomes (i27-Exosomes, i35-Exosomes). Importantly, immunotherapy with i27-Bregs or i35-Bregs suppress CNS autoimmune disease and *ex-vivo* generate exosomes also suppress disease, indicating that i35-Breg/i35-Exosome combination is effective in treating inflammatory diseases.

Steroids are first-line treatment for inflammatory diseases although second-line treatments include, calcineurin inhibitors, mTOR-inhibitors, and cytostatic agents are also used. However, these immunosuppressive therapies have significant adverse effects and are the impetus to develop safer therapies. In this review, we have described two emerging immunotherapies for CNS autoimmune diseases. As discussed, i27-Exosome and i35-Exosome immunotherapies suppress disease in mouse models of human uveitis and multiple sclerosis. However, impediment to approval of therapeutic use of exosomes is difficulty of full characterization of their cargo. These include microRNAs, lipids, carbohydrates and proteins of unknown functions.

On the other hand, Camelid-derived nanobodies have desirable attributes. They are not immunogenic in humans and their small size facilitates transport across the BBB and BRB, providing important advantages for therapeutic use in CNS diseases. The SBT-100 Nanobody is very effective in preclinical studies where it suppressed CNS autoimmune diseases with no discernible adverse effects. It holds significant promise for use in suppressing important undruggable targets inside cells, such as transcription factors like the Interferon regulatory factors and STATs that regulate critical pathways implicated in cancer and autoimmune diseases.
